# An Insufferable Business: Ethics, Nonhuman Animals and Biomedical Experiments

**DOI:** 10.3390/ani5030376

**Published:** 2015-07-22

**Authors:** Kay Peggs

**Affiliations:** Oxford Centre for Animal Ethics, School of Social, Historical and Literary Studies, University of Portsmouth, Milldam, Burnaby Road, Portsmouth PO1 3AS, Hampshire, UK, E-Mail: kay.peggs@port.ac.uk; Tel.: +44-023-9284-6093; Fax: +44-023-9284-2173

**Keywords:** anthropocentric, biomedical, business, ethics, experiments, nonhuman animals, profits

## Abstract

**Simple Summary:**

This paper explores the ways in which biomedical research that uses nonhuman animal subjects generates financial profits and gains for humans who are associated with the industry. Research establishments, scientists, regulators and persons that inspect laboratories for compliance, those associated with granting licences, companies that sell nonhuman animal subjects and that supply equipment for the research, and corporations that market the resulting products are among those that benefit financially. These profits are rarely discussed—they seem to be camouflaged by the focus of the moral convention that assumes that human health-related needs prevail over those of the nonhuman animals who are so used. The paper concludes by calling for an end to the denigration of nonhuman animals as experimental subjects who can be used as commodities for profit-maximisation and as tools in experiments for human health benefits.

**Abstract:**

Each year millions of nonhuman animals suffer in biomedical experiments for human health benefits. Clinical ethics demand that nonhuman animals are used in the development of pharmaceuticals and vaccines. Nonhuman animals are also used for fundamental biomedical research. Biomedical research that uses nonhuman animals is big business but the financial gains are generally occluded. This paper explores how such research generates profits and gains for those associated with the industry. Research establishments, scientists, laboratories, companies that sell nonhuman animal subjects, that supply equipment for the research, and corporations that market the resulting products are among those that benefit financially. Given the complex articulation of ethical codes, enormous corporate profits that are secured and personal returns that are made, the accepted moral legitimacy of such experiments is compromised. In order to address this, within the confines of the moral orthodoxy, more could to be done to ensure transparency and to extricate the vested financial interests from the human health benefits. But such a determination would not address the fundamental issues that should be at the heart of human actions in respect of the nonhuman animals who are used in experiments. The paper concludes with such an address by calling for an end to the denigration of nonhuman animals as experimental subjects who can be used as commodities for profit-maximisation and as tools in experiments for human health benefits, and the implementation of a more inclusive ethic that is informed by universal concern about the suffering of and compassion for all oppressed beings.

## 1. Introduction

The globally-prominent current neoliberal form of governance reduces everything and everyone to a commodity [1]. David Harvey comments that neoliberalization “has meant, in short, the financialization of everything” [2] (p. 3). Based in cutting public expenditure whilst increasing privatisation and entrenching decentralisation, neoliberal economics have transformed the provision of services including those associated with human health [3]. The increasing commodification of human health provision is based in the “alleged virtues of the untrammelled market, business culture and entrepreneurship” [4] (p. 24) and this market-based, profit-driven approach is central to all elements of human healthcare [5], including that which involves the use of nonhuman animal subjects in biomedical research [5]. Utility, profit and wealth undoubtedly influence choices made about nonhuman animal biomedical research [6] because experimenting on nonhuman animals is big business [7]. For example, the research and development of medicines for the pharmaceuticals industry is largely based in the use of nonhuman animal subjects [8]. Reports suggest that testing a potential medicine can involve the use of up to 800 nonhuman animals at a financial cost of over US$6 million [9]. The nonhuman animals used and the money spent are clearly thought to be worth it by the pharmaceuticals industry; worldwide the industry is worth US$300 billion, with an anticipated rise to US$400 billion within three years [10]. In this paper I centre on the profits that are made from the nonhuman animal subjects who are used in such research. I do this in order to call into question the often purely ethical rationale that is invoked to justify the use of nonhuman animals in biomedical research. I draw on Robert Garner’s [11] work on the moral orthodoxy to explore the pretext for the use of nonhuman animal beings for human health benefits. Research that uses nonhuman animals for experiments and testing is considered to be a moral issue in a number of countries. For example, in the European Union (EU) the use of nonhuman animals for research and testing of cosmetics is considered to be unwarranted and unethical and thus is illegal [12], whereas the use of nonhuman animal subjects in biomedical research is considered to be warranted and ethical if it conforms to the legal requirements [13]. The legal position, and associated moral orthodoxy, gives primacy to human-health related medical science. This primacy is found, as well, in public attitudes. There is more public acceptance of experiments that use nonhuman animal subjects if the experiments have human health objectives [14]. Because I challenge the ethics of the use of nonhuman animals in biomedical science, which is given ethical primacy, I seek to confront the assumption about the ethical legitimacy of the use of nonhuman animal experiments across the board.

In order to achieve my aims I begin with an exploration of the profit-led agenda that pervades the human concern for human health. Clinical ethics demand that nonhuman animals are first in line for much human-centred biomedical research and development, and in challenging the assumptions contained in such ethical codes I explore how the annual use of millions of nonhuman animals in biomedical research generates profits and gains for those associated with the nonhuman animal experiment industry. These profits are rarely discussed—they seem to be camouflaged by the focus of the moral convention that assumes that human health-related needs prevail over those of the nonhuman animals who are so used. Gains and profits are generated for those associated with the nonhuman animal biomedical research industry. Research establishments, scientists, technicians and other professionals who work in laboratories, regulators and persons who inspect laboratories for compliance, those associated with granting licences, companies from which nonhuman animal subjects are bought and that supply the equipment for the research, and corporations that sell the resulting pharmaceuticals and procedures are just some of those that benefit financially from the research. But required partiality to human health benefits and the claim that biomedical experiments that use nonhuman animal subjects are ethical because they conform to required clinical ethical codes and conventions conceal these revenues. I conclude that even this anthropocentric form of ethics is compromised by the prominence of profit-making for the industry and the gains that are made by researchers who use nonhuman animals in biomedical experiments.

## 2. Human Health and the Neoliberal Agenda

In this current phase of modernity, which Zygmunt Bauman characterises as “a contraption attempting to make life with fear liveable” [15] (p. 6), our fear of the unknown is eased by hope. In the case of human illness our anxious lives are made more bearable by the hope that we can predict and control a range of diseases and conditions. An inevitable consequence of the hyper-visibility of human health risks is that we feel threatened by an increasing range of maladies [16] (p. 50) even though human health has generally improved [17]. Our anxieties persist because we cannot eradicate the current array of human diseases and, even if we could, human movement across the globe has led to the resurgence of older infectious diseases and the development of newer ones [18] (p. 8). Furthermore, the spread of new strains of contagious diseases [such as pandemic influenza and Ebola] has resulted in additional worries about the development of conditions that might be resistant to known treatments [16]. Our health anxieties are exacerbated by developments in genetic research, which enable predictions to be made about our individual probability of developing specific genetic conditions [19]. Although this “era of personal genomics” [20] means that predictive and precision medicine and treatments can be used, such predictions can induce fear in those of us who are told that we hold the genetic markers of a dreaded disease [21]. In this “age that is *guided* by science” [22] (p. 15) we hope that scientific research and expert systems [23] (p. 115) will help us stave off feared and unwanted threats to our health. This dependence on medicine is exacerbated by our fear of ageing and death, which have become medicalised processes [24]. Atul Gawandi observes that “Lacking a coherent view of how people might live successfully all the way to their very end we have allowed our fates to be controlled by the imperatives of medicine, technology, and strangers” [24] (p. 8). We have a great deal of confidence in medicine [25] and undoubtedly human health has improved as a result of medical innovations. But scientific breakthroughs have not given us unconstrained control over our health futures, though the calculation of the probability of a health hazard occurring is “the next best thing to (alas unattainable) certainty” [15] (p. 10). 

Our concern about our health is evidenced by the amount we spend every year on human health care. Health care is big business. For example, the USA is reported to have the highest health spending in the world, in 2012 equivalent to nearly 18 percent of its Gross Domestic Product (GDP) [26]. In the UK the corresponding figure was 9.6 percent of GDP [26]. Increased health spending is said to deliver better services to humans who are in need of those services. In the UK, health provision is claimed to be “continuously evolving with the aim to deliver improved services to patients” [27] (p. 11). But this “continuously evolving” system comprises a mix of market and non-market sectors [27]. Like in the USA [28] (p. 14), health care in the UK is characterised by “medical neoliberalism”, which is based in individual responsibility for health and the notion that private providers in the market sector offer medical services that are of higher quality than those provided by the non-market sector [29]. So, along with welfare, education and social security, the focus of health care is on free markets and free trade [26] (p. 14). Free market delivery mechanisms (which are the professed health care reforms we are familiar with hearing about in the UK) centre in a for-profit system [29] (p. 83). This neoliberal for-profit health care system means that a growing share of the global spending on health care, amounting to over US$5 trillion, is being shifted from the public to the private sector [30]. Elemental to this is the medicalisation of an increasing range of human conditions, to which pharmaceutical companies respond by charging high prices for an increasing range of drugs [31] (p. 24). But those who are least in a position to pay are often those who are in most need of health care, for example the oldest and the youngest citizens, those who live with chronic illness or disabilities, and those who are living in poverty [30]. Even in the wealthy USA, high prices are making health care too expensive for many [32]. Dr. Pamela Hartzband and Dr. Jerome Groopman lament, “Patients are no longer patients, but rather customers or consumers” [33] (p. 102).

There is a lot of money to be made out of the customers and consumers of services and products for human health and, where there is little money to be made pharmaceutical companies, for example, are less likely to innovate [34]. The desire to maximise profits has been pointed out in current news media reports about the Ebola virus. In Guinea, Liberia and Sierra Leone there have been a total of 26,044 “confirmed, probable and suspected cases” of Ebola and at least 10,808 people in those countries have been killed by the virus [35]. In Europe there have been two reported cases (one in Spain and one in the UK) and in the USA there have been four cases of Ebola, and one reported death [35]. At the time of writing there is no vaccine against the virus [36]. Erica Etelson [37] suggests a reason for this. She refers to Margaret Chan’s (Director of the World Health Organization) assertion that the pharmaceutical industry’s failure to develop a vaccine up to now is because “A profit-driven industry does not invest in products for markets that cannot pay” [37]. Etelson [37] infers that, had the Ebola virus been detected in the USA sooner, a vaccine would already be available. She concludes that “Big Pharma’s greed isn’t some kind of aberration; it’s an inherent feature of free-market capitalism: A capitalist system, by design, puts profits over people” [37]. This emphasis encourages a perspective on “benefits” that is oriented towards benefits *as profits* rather than on benefits *as improvements to human health* [31].

This is a disgrace, but it is not the only disgrace. This free-market health system ensures that the human health industry not only redefines benefits as profits and promotes profits *over* human beings, it makes profits *from* the use of nonhuman animals. These financial profits and gains are often occluded by the focus on the human health benefits that are purported to make such experiments ethically justifiable.

## 3. Research Ethics and Biomedical Experiments

The sought after utopian future based in the human ability to prevail over our bodily limitations [38] is based in a commodified, dystopian past, present and likely future of misery and suffering for millions of nonhuman animals who are used in biomedical experiments [16]. However, this cost to nonhuman animals is considered to be warranted if the human health benefits that ensue are important enough to justify the suffering caused [11]. P. Michael Conn and James V. Parker argue that “virtually every major medical advance of the last century is due, in part, to research with animals” [39] (p. 158). The human health benefits are summarised by Professor Robert Winston on the UK-based *Understanding Animal Research* (UAR) alliance website [40]. He states that, “Animal research has advanced the treatment of infections, helped with immunisation, improved cancer treatment and has had a major impact on managing heart disease, brain disorders, arthritis and transplantation” [40]. In addition, UAR announces a range of future human health possibilities that could result from such experiments, such as the treatment of chronic diseases [41]. Although there is no legal requirement for nonhuman animals to be used for fundamental biomedical research there is a legal requirement for nonhuman animal subjects to be used in the development and testing of medicinal drugs and vaccines [42] which, the Association of the British Pharmaceutical Industry among others declares, is “to ensure patient safety” [43]. This form of human protection was defined in the Nuremberg Code.

The Nuremberg Code, which has served as a foundation for ethics in clinical research, comprises a series of research ethics principles for human experimentation that were drafted at the end of the Doctor’s trial in Germany in 1947 [44]. It is in Item 3 of the code that reference is made to nonhuman animals. This item states that an experiment that uses humans “should be so designed and based on the results of animal experimentation and a knowledge of the natural history of the disease or other problem under study, that the anticipated results will justify the performance of the experiment” [45]. Further protocols have followed, such as the Helsinki Declaration, which reinforce the requirement that nonhuman animal experiments should precede human experiments [46]. This requirement is repeated in national and international laws, codes, and declarations. Such protocols are designed to protect against human experimentation atrocities like the Tuskegee Syphilis Study, Human Radiation Experiments in America, Nazi Experiments in Germany, and the compulsory sterilization programme in India [47]. But such atrocities continued and persist. For example, the Tuskegee Syphilis Study continued until 1972 and, in terms of current practices, Mohammed Imran and colleagues observe that “Still unethical studies are found in developing countries. Studies such as experimental anticancer drugs in 24 cancer patients without adequate prior animal testing and informed consent in Kerala” [47] (p. 203). The lack of informed consent is shameful, dangerous, and unethical. Leaving aside, for a moment, the ethical issues associated with using nonhuman animal subjects in such experiments, there is no consensus about the adequacy of protection that is offered to humans by the prior testing of drugs on nonhuman animal subjects.

The claims that biomedical experiments that use nonhuman animals protect humans are contentious. Although a 2011 poll of nearly 1000 biomedical scientists undertaken by Daniel Cressey (for the journal *Nature*) found that more than 90 percent “agreed that the use of animals in research is essential” [48], alternative scientific opinions identify problems, and indeed risks, associated with the prediction of human health outcomes on the basis of nonhuman animal experiments [49]. Probably the most obvious example is that of the drug Thalidomide, which was first prescribed to pregnant women in the 1950s to alleviate nausea. The drug led to devastating foetal effects in humans. After it was withdrawn tests in pregnant mice, rats, and guinea pigs led to no negative foetal effects, though testing in New Zealand white rabbits led to similar devastating foetal effects as found in humans [50] (p. 125). Another reported problem lies in the effective treatments for humans that would not be available if nonhuman animal tests were relied upon [7]. For example, some cancer drugs that are used as effective treatments for human cancers were ineffective in treating cancer in mice [7] (p. 17). Concerns have also been raised about the scientific rigour of some experiments. For example, Pandora Pound and her colleagues argue that many studies that use nonhuman animals are of “poor methodological quality” [51] (p. 517) which means that the research is “wasted because it is poorly conducted and not evaluated through systematic reviews” [51] (p. 514). Ray Greek and colleagues conclude that “the requirements for animal testing found in the Nuremberg Code were based on scientifically outdated principles, compromised by people with a vested interest in animal experimentation, serve no useful function, increase the cost of drug development, and prevent otherwise safe and efficacious drugs and therapies from being implemented” [52] (p. 1). As we have seen, Michael Conn and Parker disagree [39] (p. 158). This is a vital difference of scientific opinion but the fundamental issue is not the efficacy of the use of nonhuman animal subjects in biomedical experiments it is the ethical legitimacy of nonhuman animal experimentation [5].

The assertion that prior testing on nonhuman animals has ethical legitimacy is doubted by many, including doctors [53] and patients [54]. For example, a study in Sweden [54] suggests that although many researchers support nonhuman animal biomedical research broader support for such research is not shared to an equal degree with human patients, who are the intended end-users and beneficiaries of such research. This leads the authors, Malin Masterton and colleagues, to advise that “The moral basis for using animals in research needs to be further discussed by all stakeholders” [54] (p. 24). The overlooked stakeholders, the nonhuman animals who are used in biomedical research, do not have a voice. We cannot deny that Jacques Derrida is right; nonhuman animals do suffer [55] (p. 28) and ethical consideration of their suffering should have no prior limits or constraints [56]. In what remains of this paper I offer a challenge to the anthropocentric ethics that permits nonhuman animal experiments to take place by bringing to light the profits and gains made by those who are associated with the industry, profits that are camouflaged by the position that human-health needs prevail over the wellbeing of the nonhuman animal subjects who are so used. Ultimately I challenge the ethical legitimacy of the use of nonhuman animal subjects in biomedical research.

## 4. Nonhuman Animals, Ethics and Biomedical Experiments

A conservative estimate puts the number of living vertebrate nonhuman animals who are used annually in experiments worldwide at 115.3 million [57] (p. 327). The majority of countries (79%) do not publish statistics so it is difficult to obtain accurate data [57] (p. 327). For example the People’s Republic of China, Egypt, Iran, India, and Thailand do not provide statistics about the number of nonhuman animals they use. However, on the evidence available Katy Taylor and her colleagues estimate that the United States, Japan, China, Australia, France, Canada, United Kingdom, Germany, Taiwan and Brazil use the highest numbers [57] (p. 327). The figures for most countries cover mainly vertebrate nonhuman animals (who are considered to feel pain); there are many more invertebrates [58] (and some vertebrates) who are not included in the statistics as they are not considered to be “animals” for purposes of “protection” [59]. For example, according to the USA Department of Agriculture, Animal and Plant Health Inspection Service, in the Fiscal Year 2010 over 1.13 million nonhuman animals were used in experiments in the USA [60] but the USA Federal Animal Welfare Act excludes “cold-blooded” nonhuman animals and rats, mice, birds, reptiles, amphibians, and nonhuman animals used in agricultural experiments. Rats and mice comprise the overwhelming majority of all laboratory subjects used [61]. The figure of 11.5 million nonhuman animals who were used in experiments in the European Union (EU) in 2011 [62], does include mice and rats but, with the exception of some cephalopods, does not include invertebrate species. As in many other countries the EU statistics also exclude “the maintenance of breeding genetically modified…animals, animals killed solely to provide tissue, “surplus” conventional animals bred but then killed, and animals exploited in behavioural research, including the marking of “wild” fish” [63]. In 2013 over four million of the stated total number of nonhuman animal subjects used in the EU were used in experiments in the UK (4,017,758 nonhuman animals used in 4,121,582 experiments) an increase of 0.3 percent (+11,600 procedures) on the previous year [64]. Again most invertebrate species were excluded from this figure. The UAR acknowledges that for the UK “The number of animals used in research has risen in recent years” but, the organisation suggests, “This is for a variety of reasons, not least because more research is being carried out to understand, prevent and treat human and animal diseases, and to protect the environment” [65]. Although statistical data provide a measure of the quantitative scale of the pain and suffering caused, such data do not call attention to the experiences of suffering of individuals [66] and thus can serve to distance us from understanding the lived experiences of individual nonhuman animal subjects who are used in biomedical research [67]. Individual nonhuman animals are subjected to “procedures against their own individual interests, including those that involve the deliberate infliction of suffering, harm, and/or death” [63].

Nonhuman animal subjects are used in both biomedical and non-biomedical research. Non-biomedical research includes experiments to determine the effects and safety of a range of products, processes, and events such as those associated with warfare, accidental damage, food processing and cosmetic enhancements [57]. The distinction “biomedical” and “non-biomedical” is important, notwithstanding how ambiguous it might be, in terms of industry justifications and public views about the veracity of nonhuman animal experiments. Regarding public support, it is usually reported that there is less support for research that uses nonhuman animals if the research is not seen as necessary for human health benefits. For example, a 2012 Ipsos/Mori poll in the UK revealed that 66 percent of those who conditionally support experiments that use nonhuman animals only do so if the experiments are for medical research purposes “where there is no alternative” [68] (p. 7). This is because (at least some) nonhuman animals are considered to have moral standing [11].

A being has moral standing if she or he can be morally wronged [69]. Possession of moral standing means that the being concerned should be given moral consideration. There is considerable discussion about the source of moral standing [69] and the foundations are complex and nuanced. It is not possible to do justice to the discussion here. However what is clear, fundamentally, is that all humans have moral standing and have *equal* moral standing, which means that often we hear what Richard J. Arneson refers to as “platitudinous statements” [70] (p. 103) that humans should not be subjected to discrimination. Platitudinous because, despite there being equal moral standing among all humans, many humans and groups of humans are subjected to discrimination. In contrast, for nonhuman animals discrimination is permitted and required, even where their moral standing is recognised. A continuum of recognition of the moral standing of nonhuman animals which draws out the differences has been developed by Garner [11]. At one end of this continuum is the idea that nonhuman animals have no moral standing because, for example, they are deemed to lack sentiency. In the centre of the continuum is the current moral orthodoxy, which summarizes the existing UK position on the moral standing of “protected” nonhuman animals. Garner explains that under the terms of the “moral orthodoxy” it is expected that the interest that nonhuman animals have in not suffering can be overridden for what is considered to the greater good of humans [11] (p. 15). At the other end of the continuum there are challenges to both the granting of no moral standing to nonhuman animals and to the moral orthodoxy because at this end nonhuman animals are granted moral equality with humans [11] (p. 18).

The current moral orthodoxy is entrenched in biomedical research as “it is widely held that partiality to human interests is not only defensible, but obligatory” [71] (p. 245). Although many biomedical researchers seek to fulfil the moral imperative of the 3Rs (replacement, reduction, and refinement) and aspire to eliminate the use of nonhuman animal subjects [57], harm to nonhuman animal subjects in laboratories “is counted as a small thing in comparison with the research work undertaken” [63] (p. 67). The required partiality to human interests is based in the widely held view that nonhuman animals have some moral status but that humans are morally superior. This serves to support the claim that biomedical research that uses nonhuman animal subjects and which conforms to the legal requirements is unquestionably ethical. But much of the research that goes on in the biomedical sphere is morally illegitimate even within the limitations of the moral orthodoxy [11]. The moral legitimacy is compromised by the profit-making nonhuman animal experiment industry and the gains that are made by researchers who use nonhuman animals in biomedical research.

## 5. Research Funding

Biomedical research is funded by governments, private companies and organisations, and by donations from individuals and charities. In the UK, biomedical research receives millions of pounds of government funding. For example, in 2012 the UK Government announced “the Biomedical Catalyst….an integrated £180m funding programme to support the development of innovative solutions to healthcare challenges by both SMEs (Small and Medium-Sized Enterprises) and academics across the UK” [72] (p. 15). Private awards and grants augment the amount that is available for such research. In the UK the biggest investor is the pharmaceutical industry, which spends £12 million a day on such work, representing 70 percent of the total funds [73] (p. 5). Much of this money is directed to experiments that use nonhuman animal subjects. Charitable donations make additional and significant contributions to the funding of such experiments. Out of a total income of £214 million for 2009/10 the *British Heart Foundation* allocated £48.4 million to the funding of research that involved nonhuman animals [74]*.* In the same year the UK *Alzheimer’s Society* assigned £700,000 of its annual income of £58.7million to such work and in 2010 *Parkinson’s UK* apportioned £8 million of its total income of £21million [74]. A spokesperson for the charity *Cancer Research UK* commented that “We have strict ethical policies in relation to animals and follow rigorous government guidelines to ensure that animals are only used where there’s no alternative” [74]. *Cancer Research UK* allocated £334 million of its annual 2009/10 income of £446 million [74]—that is nearly twice as much as the UK government set aside in 2012 for its “Biomedical Catalyst” [72] (p. 15).

The costs of biomedical research and development are escalating [75]. Much of the money available is used to fund a range of organisations that undertake nonhuman animal based biomedical research. A great deal of such research is carried out in universities. Universities in the UK are part of an “academic political economy” that is characterized by decreasing government funding so “the application of corporate business practice in universities has been widespread, to ensure their survival” [76] (p. 293). Although 24 of the 64 projects that are receiving funds from the “Biomedical Catalyst” are university-led [72] (p. 15), funding from private organisations outstrips that secured from government sources so gaining external funding, (such as from the pharmaceutical industry and charitable donations) is crucial. Principal biomedical research universities in the UK, such as Edinburgh, Oxford and Cambridge, devote vast sums of money to research that uses nonhuman animal experiments [77]. There is a greater sum of federal funding in the USA. According to the People for the Ethical Treatment of Animals (PETA) [78] the USA National Institute of Health (NIH) allocates 40 percent of its annual research budget to research that uses nonhuman animal subjects. Using this figure they calculate that NIH spent more than $16 billion on such experiments in 2010 alone [78]. Substantial annual increases in health-related research and development funds are demanded by biomedical researchers who argue that such increases are “essential to maintaining our momentum of progress in the battle against disease” [75]. Similar demands are being made in the UK and across the world. But the “battle against disease” is not the only awaited feature of the gaining of such funds.

Biomedical researchers in academia rely on grants to fund their research not only to fund research but because securing a research grant enhances individual academic reputations and enhances the reputation of the university at which they are employed [76]. Gaining research funding is a core component of evaluations for promotion [79]. Jean Swingle Greek and Ray Greek argue that “Conducting animal experiments is a convenient and highly effective way for these researchers to gain career prestige and job security, and for the universities who employ them to obtain lucrative research grants. There is a *quid pro quo* relationship between research institutions and those giving the grants” [7] (p. 25). In biomedical research the paradigm of nonhuman animal experiments is the accepted pattern for research and “Once a pattern of animal experimentation becomes the accepted mode of research in a particular field”, Peter Singer notes “The process is self-reinforcing and difficult to break.” [80] (pp. 90–91). There is more money available for research and development that involves the use of nonhuman animal subjects than for research that explores alternatives. The search for alternatives is often based in the 3Rs, first advanced by W. M. S. Russell and R. L. Burch in 1959 [63] (p. 43). Although the UK government makes funds available for work on alternatives [81] (p. 27) it stresses that it “recognises that the carefully regulated use of animals plays an essential role in scientific research, particularly in ensuring new medicines are safe and effective” [72] (p. 40). Humane Society International states that “For 2011/2012, the government gave just £5.46 million to the National Centre for the Replacement, Refinement and Reduction of Animals in Research, with roughly one-third spent on research to replace animals. This is compared to an overall annual UK science budget of £4.6 billion” [82]. Consequently, individuals and corporate bodies who “have career advancement, prestige, and the bottom line to look after, protect the animal experimentation industry under the guise of protecting human health” [7] (p. 24).

## 6. The Business “Animal Model”

The UK pharmaceutical industry alone invests vast sums of money in research and development. Across the EU in 2010 the pharmaceutical industry invested €27 billion in these activities [73] (p. 5). The UK pharmaceutical industry reports that 37 percent of current sales return is spent on research and development [73] (p. 5). When we recall that the UK industry spends £27 million daily on such work (see above) we gain an insight into the scale of their total sales return. An indication of the profits made from pharmaceuticals can be gleaned from individual company returns. For example, the UK-based pharmaceuticals company *GlaxoSmithKline* reported a pre-tax profit of £548 million for the three months to the end of September 2014 which, although considerably down from the figure of £1.4 billion for the same period in 2013, was reported to be better than expected [83]. Shareholders benefitted as “shares rose 4% after its third-quarter results beat expectations and it pledged to return an additional £4bn to shareholders via a special share scheme” [83]. Much of the research and development undertaken by *GlaxoSmithKline* involves experiments that use nonhuman animal subjects. Although the company does not provide the statistics it is evident that nonhuman animals subjects are used as it reports that the number used in 2013 was down by 10% on that for 2012 [84]. Additionally, the company argues that “Animal testing is not the cheap option—our animals are fed, housed and looked after by qualified professionals throughout their lives. A veterinarian is also on site or on call 24 h a day, seven days a week” [84]. In the UK, veterinary practitioners and Named Animal Care and Welfare Officers (NACWOs) are employed in laboratories where nonhuman animal subjects are used in experiments [67]. Their role is to recognise and monitor the suffering of nonhuman animals in the laboratory with a view to improving their welfare [85]. This brings me to the money that can be made by those who sustain the use of nonhuman animals in biomedical experiments.

With at least 115.3 million nonhuman subjects used in experiments across the world every year there is a great deal of money to be made out of the breeding and selling of nonhuman animals to laboratories. *Charles River* is one of the suppliers of nonhuman animals to the global industry. The list of nonhuman animals the company supplies is extensive. Mice and rats are used in vast numbers in biomedical experiments, not least because they are thought to react to medication in similar ways to humans [86] and because they are relatively inexpensive to buy [87]. A three week old Swiss pigmented mouse, who is marketed as being “for general purpose”, is sold by *Charles River* for US$10.10 [if he is male] and for US$10.95 (if she is female) [88]. The “genetically engineered” mouse who is sold under the label 11BHSD2 is bred in the USA and is promoted by *Charles River* as being “Ideal for cardiac hypertrophy, heart failure” [88]. Depending on “types” and age an individual mouse costs between $32 and $181, with additional charges of $10 for each additional week of age [88]. In 2013 the company’s total revenue was US$1.17 billion [89]. Rats and mice who are bred for use in the laboratory have been subjected to decades of selective breeding [90] (p. 22), and the genetic “engineering” of nonhuman animals to match the needs of researchers who focus on specific conditions is now mainstream. When such genetically modified nonhuman animals were developed and began to be sold as commodities on the market substantial profits were made by the humans and businesses involved. Huge profits continue to be made. The most familiar example is OncoMouse^®^, a transgenic mouse who was developed in 1984 by Harvard to have a predisposition to cancer. The comprehensive commodification of the mice is evidenced by the news that OncoMouse^®^ was named “Product of the Year” in 1988 by *Fortune* [91]. Not a mouse, a “product”. A great deal of money continues to be made out of the mice. Du Pont, the company that subsequently owned the patent for these living beings, imposed “hefty fees and restrictions” on the use of OncoMouse^®^ [92]. The patent for OncoMouse^®^ remains in dispute. Douglas Hanahan and colleagues argue that “Such use of technology patents as end products to generate revenue, rather than as means to develop products, is a socially controversial if not uncommon business practice that is applied to research tools in biology and medicine” [92] (p. 2268).

Laboratories that use nonhuman animal subjects as experimental tools need to buy a range of equipment to facilitate the experiments and to attend to the welfare of the nonhuman animals involved. As we have seen, *GlaxoSmithKline* remarks on the heavy financial costs of the additional resources that are required for its nonhuman animal biomedical experimentation programme. *GlaxoSmithKline* makes vast profits while at the same time providing paid employment for related employees and enabling profits to be made by companies that supply them with the nonhuman animals and the equipment they use. Paradoxically, policies aimed at improving the welfare of nonhuman animals who are used in laboratories have the potential for increasing the profits of suppliers as laboratory equipment must conform to statutory requirements [5]. The equipment that is used in such research includes that which is associated with housing, feeding, experimenting on and the killing of nonhuman animal subjects, The catalogues of suppliers include advertisements for cages, restraints and guillotines. The Canada-based company *Lomir Biomedical* is a supplier of a range of equipment for such research. The company markets a variety of products and strives to invent new ones that can be marketed as responding to the changing needs of biomedical scientists. For example, the company states that it has “identified a need for a range of undershirts for laboratory animals as scientific procedures become much more refined. Undershirts are an effective means of securing electrodes, connectors, Fentanyl patches or any piece that needs to be kept in contact with skin.” [93]. One dog undershirt costs either US$50.99 or US$54.11, depending on how many are ordered [93]. The company has an annual revenue of US$4,695,040 [94].

The multi-million dollar industry in nonhuman animal experiments is thriving [7]. Those who supply nonhuman animals to research laboratories, along with the apparatus needed to house, feed, and experiment on nonhuman animal subjects have a vested interest in sustaining the nonhuman animal experiment paradigm [7]. The profits compromise the moral legitimacy of nonhuman animal biomedical experiments even with the limited gaze of the moral orthodoxy.

## 7. Conclusions

In this paper I have sought to examine the ways in which biomedical research that uses nonhuman animal subjects generates financial profits and gains for humans who are associated with the industry. My aims have been to make evident the often occluded economic benefits that are associated with such research and, ultimately, to challenge the moral orthodoxy that permits nonhuman animal experimentation to take place at all. The moral orthodoxy requires that biomedical experiments that use protected nonhuman animals should conform to a cost-benefit analysis, where the benefits to humans are deemed to be important enough to justify the costs (that is the suffering) to the nonhuman animal subjects who are so used [11]. The human benefits that are considered to be important enough mainly are associated with the advancement of knowledge and the development or toxicity testing of clinical interventions [63]. Even in the current neoliberalised medical system that encourages a perspective on “benefits” that is oriented towards benefits as profits rather than on benefits as improvements to human health [31] the moral orthodoxy is not satisfied as the production of economic benefits is not important enough for nonhuman animal suffering to take place under its terms.

The established paradigm on nonhuman animal biomedical experiments is based in the argument that nonhuman animal experiments have utility because they have led to progress in the treatment of human illnesses and offer safeguards in terms of patient safety. The moral orthodoxy is fulfilled by the established paradigm if the legal requirements are adhered to and are satisfied. From this perspective the financial benefits that accrue, and indeed the money that is spent in the first place, are side issues as the main consequences of nonhuman animal biomedical experiments are the benefits to humans that ensue by means of the development of new medicines or vaccines, improved diagnosis or better methods of toxicity testing [8]. However, there is no consensus about this as there are those who argue that human health does not benefit as humans are harmed by nonhuman animal biomedical experiments because they cannot predict responses in humans [63]. From this perspective the money is not well-spent as vested interest groups profit financially at the expense of human patients and thus the benefits are not health-related, rather the benefits come in the form of the profits gained [7].

So, there is considerable doubt that some of what happens in the biomedical sphere is morally legitimate even within the limitations of the moral orthodoxy [11]. Given the differences in opinion about the efficacy of nonhuman animal experimentation and the complex articulation of ethical codes, enormous corporate profits that are secured and personal returns that are made, the moral orthodoxy is compromised. In order to address this, within the confines of the moral orthodoxy more could to be done to extricate the vested financial interests from the human health benefits. The focus could be on obligatory transparency and accuracy of numbers of nonhuman animals used, with a calculation that includes all nonhuman animals who are used and who are squandered, a scrupulous analysis of the effectiveness of nonhuman animal experiments and an openness about the financial profits that are made. These determinations would make evident the efficacy, or not, of the use of nonhuman animal subjects in biomedical experiments in tandem with the often occluded economic benefits that are associated with such research. Given that nonhuman biomedical experiments are given ethical primacy within the narrow scope of the moral orthodoxy, without such determinations the moral legitimacy of the use of nonhuman animal subjects in all experiments will continue to be questioned. However, these determinations remain restricted by an anthropocentric standpoint. They would not address the costs to the nonhuman animal subjects who are used in biomedical experiments because these costs would continue to be permitted within the limitations of this anthropocentric moral orthodoxy.

I want to close with a challenge to the moral orthodoxy, a challenge that seeks to address the fundamental issues that should be at the heart of human actions in respect of the nonhuman animals who are used in experiments [67]. Such a challenge does not focus on the efficacy of experiments or on transparency about numbers used and profits made, rather it centres on the implementation of a more inclusive ethic that is informed by universal concern about the suffering of and compassion for all oppressed beings [95]. This would affect a fundamental shift in the way we view nonhuman animals, away from an anthropocentric approach to a way of looking at the world from the standpoint of nonhuman animals with the goal of ending the denigration of nonhuman animals as experimental subjects who can be used as commodities for profit-maximisation and as tools in experiments for human health benefits. At the time of writing a petition (signed by 1.2 million people) has forced Ministers of the European Parliament to debate a ban on nonhuman animal experimentation [96]. With the stated aim of protecting humans and nonhuman animals, the petition demands legislation that will see the abolition of “animal experimentation and instead makes compulsory the use—in biomedical and toxicological research—of data directly relevant for the human species” [97]. Such a shift in legislation, and in the allocation of funds, would affect moral progress by advancing the wellbeing of nonhuman animals, well-being that is based in freedom from human-induced pain and suffering.
